# Development and Initial Assessment of a Low-Cost Skin Tear Management Task Trainer for Paramedic Education

**DOI:** 10.7759/cureus.117492

**Published:** 2026-10-05

**Authors:** Anjali Jagannathan, Jessica Clark, Katherine Robertson, Nicole Gilchrist, Adam Dubrowski

**Affiliations:** 1 Health Sciences, Ontario Tech University, Oshawa, CAN; 2 Paramedicine, Region of Durham Paramedic Services, Whitby, CAN

**Keywords:** additive manufacturing, digital technologies, simulation-based education, skin tear management, task trainer, wound care

## Abstract

Inappropriate management of skin tears can result in pain, delayed healing, infection, and reduced mobility. There is growing interest in expanding the paramedic scope of practice to include skin tear management. However, a lack of scalable, low-cost task trainers poses barriers to the implementation of structured training initiatives. Hence, this technical report describes the development of a scalable, low-cost skin tear management task trainer for paramedic education using digital technologies and additive manufacturing, and provides an initial assessment of its perceived acceptability.

Three skin tear management task trainer prototypes (type 1, no skin loss; type 2, partial skin loss; type 3, complete skin loss) were co-developed by a team of researchers and an advanced care paramedic. The development process consisted of two iterative rounds of prototyping, testing, and refinement, after which 14 type 1 task trainers were fabricated, integrated into a paramedic training initiative, and assessed across three domains, including (1) self-efficacy, (2) realism, and (3) educational value. Quantitative and qualitative data were collected and analyzed.

Approximate fabrication time per unit was 20-30 minutes of active labor and 80-90 minutes of curing, depending on the skin tear type. The total cost of the materials used to fabricate the task trainer was 35.29 Canadian dollars (including taxes). This amount excludes capital equipment, including the three-dimensional printer, computer-aided design and slicing software, and reusable molds and templates, which are amortized across multiple task trainers.

Participants rated the task trainer highly for perceived self-efficacy (4.68/5), realism (4.64/5), and educational value (4.85/5). These scores reflect participants’ perceptions rather than objective measures of skill acquisition. Qualitative data yielded three descriptive categories: (1) visual and tactile realism, (2) pathological realism, and (3) procedural complexity and adaptability. Overall, both quantitative and qualitative data indicated that participants perceived the task trainer as an acceptable training tool.

This technical report demonstrates the feasibility of developing a scalable, low-cost skin tear management task trainer using digital technologies and additive manufacturing. Future work will focus on standardizing skin flap thickness and developing task trainers with varying skin tear shapes and sizes, anatomical locations, wound presentations, and levels of procedural complexity. Next steps involve implementing the task trainer broadly across paramedic training initiatives and formally evaluating its impact on procedural skill acquisition and maintenance, confidence, and decision-making abilities.

## Introduction

Skin tears are traumatic wounds in which the epidermis separates from the underlying dermis, often resulting in a partial or full-thickness wound with a friable, viable skin flap [1]. Skin tears can be classified as type 1 (no skin loss), type 2 (partial skin loss), or type 3 (complete skin loss), according to the International Skin Tear Advisory Panel (ISTAP) Skin Tear Classification System [2]. They are most prevalent among infants, older adults, and individuals with fragile skin [3], and are particularly common among hospital inpatients and residents of long-term care facilities [4]. Skin tear management may include cleaning and irrigating the wound to remove dirt and debris, reapproximating the skin flap to cover the wound, and applying a dressing [5]. These procedures require technical competence and procedural accuracy to preserve viable skin flaps, minimize pain, prevent infection, and support healing and mobility [5].

Traditionally, skin tear management has been performed by clinicians in hospital emergency departments, long-term care facilities, or community healthcare services, contributing to delays in treatment, increased patient transfers, and added strain on already overstretched healthcare systems [6]. As healthcare systems seek to improve efficiency and responsiveness, there is growing interest in shifting selected procedures, including skin tear management, into prehospital environments, where trained paramedics can provide earlier intervention [7]. Expanding the paramedic scope of practice to include skin tear management can improve treatment efficiency and reduce the reliance on hospital-based care. However, this expansion requires structured training initiatives that reflect the clinical and operational realities of prehospital care, while providing opportunities for hands-on procedural skills practice.

Simulation-based education (SBE) offers a safe and controlled environment in which paramedics can acquire and maintain skin tear management procedural skills, while also enhancing confidence and decision-making abilities [8]. Despite its potential, implementing simulation-based skin tear management training initiatives in paramedic education remains challenging, largely due to a lack of scalable, low-cost task trainers, which are physical models used for hands-on practice of specific clinical procedural skills [9]. Commercial skin tear task trainers, such as the Skin Tear with Flap trainer developed by Techline Trauma (Horsham, PA, USA) priced at 146.61 Canadian dollars (CAD) [10], may be prohibitively expensive, limited in anatomical realism, and inflexible in design, restricting their adoption across diverse training initiatives. These barriers are particularly pronounced in rural, remote, and resource-constrained settings [11].

Digital technologies and additive manufacturing (AM), such as three-dimensional (3D) printing and silicone modeling, present a promising solution to these barriers. Previous studies have demonstrated the utility of digital technologies and AM for producing scalable, low-cost task trainers to support SBE across diverse health professions training initiatives [12,13]. Such methods enable task trainers to be redesigned and customized to reflect diverse patient populations and accommodate program and learner-specific needs, while also adhering to budgetary constraints. Consequently, harnessing digital technologies and AM to develop skin tear management task trainers may facilitate the broader implementation of simulation-based training initiatives in paramedic education.

Hence, this technical report aims to (1) describe the process of developing a scalable, low-cost skin tear management task trainer for paramedic education using digital technologies and AM, and (2) assess its perceived acceptability as a training tool.

## Technical report

Context

The task trainer described in this technical report was developed in collaboration with Region of Durham Paramedic Services (RDPS; Whitby, Ontario, Canada). Previously, opportunities for hands-on skin tear management training within RDPS were limited, highlighting the need for structured training initiatives to support procedural skill development. RDPS is currently leading the development and implementation of a skin tear management training pathway aimed at supporting procedural skill acquisition and maintenance, confidence, and decision-making abilities among frontline and community paramedics. The pathway has been designed for broader implementation across diverse paramedic education programs and academic training pathways across Eastern Ontario. However, a scalable, low-cost skin tear management task trainer was identified as necessary to support the translation of theoretical knowledge into hands-on practice.

Inputs

Personnel

The task trainer was co-developed by two teams with complementary expertise, referred to in this technical report as the research team and the clinical team. The research team consisted of two researchers with expertise in SBE, digital technologies, and AM (AJ & AD) from the maxSIMhealth laboratory, a research laboratory located at Ontario Tech University in Oshawa, Ontario, Canada. The clinical team included an advanced care paramedic from RDPS with 20 years of experience (JC). The task trainer was subsequently assessed by frontline and community paramedics from RDPS.

Equipment and Materials

Table 1 shows the equipment and materials used to manufacture the task trainer.

**Table 1:** Equipment and materials used to produce the task trainer. 3D, three-dimensional; PLA, polylactic acid.

	Equipment and materials
1	Ultimaker S5 3D printer (Ultimaker B.V., Utrecht, The Netherlands)
2	Ultimaker Cura 3D printing software (Ultimaker B.V., Utrecht, The Netherlands)
3	Fusion360™ (Autodesk Inc., San Rafael, CA, USA)
4	UltiMate™ polylactic acid (PLA+) (Hamilton, ON, Canada)
5	Ecoflex™ 00-20 FAST silicone (Smooth-On, Macungie, PA, USA)
6	Ecoflex™ 00-35 FAST silicone (Smooth-On, Macungie, PA, USA)
7	Silc-Pig™ coloring (Smooth-On, Macungie, PA, USA)
8	Ease Release 200 (Sculpture Supply Canada, Toronto, ON, Canada)
9	Power mesh (Green Brook, NJ, USA)
10	Aleene's Fabric Fusion Stiffen Quik Pump Spray Adhesive, Quick Drying, Stiffening Fabrics (ILoveToCreate, Los Angeles, CA, USA)
11	Cozylkx Upholstery Foam (Cozylkx, China)
12	MAYKU Form Box Clear Sheets (Bermondsey, London, UK)

Process

Development of the Skin Tear Management Task Trainer

The development process consisted of two iterative rounds guided by three constraints established by the clinical team: (1) the design must be realistic and adaptable to reflect diverse patient populations and accommodate learner and program-specific needs; (2) the task trainer must be durable and allow for repeated use over time; and (3) the process must adhere to a design-to-cost approach [14], optimizing costs while maintaining realism, adaptability, and durability.

In each round, prototypes were developed by the research team and evaluated by the clinical team using the think-aloud protocol (TAP) [15], which involves verbalizing thoughts while performing tasks to reveal underlying cognitive processes. This approach is commonly employed in research and quality improvement initiatives to gain insights into decision-making, problem-solving strategies, and product design issues [15]. During testing, the research team took notes in a Google document (Google, Mountain View, CA, USA), which were subsequently analyzed to guide refinements to the task trainer.

Round 1

Prototyping: In round 1, the research team produced three prototypes, each representing one of three skin tear types (type 1, no skin loss; type 2, partial skin loss; or type 3, complete skin loss) according to the ISTAP Skin Tear Classification System [2], using digital technologies and AM.

A previously developed advanced intraosseous (IO) infusion task trainer was repurposed to serve as the foundation for the skin tear management task trainer [16]. The IO task trainer was developed using digital technologies and AM and consists of three components: (1) a 3D-printed leg with a removable tibia bone and foam pads to simulate adipose tissue; (2) a replaceable silicone skin overlay; and (3) a 3D-printed stand [16].

The leg, tibia bone, and stand were digitally designed using Fusion360™ (Autodesk Inc., San Rafael, CA, USA), sliced using Ultimaker Cura 3D printing software (Ultimaker B.V., Utrecht, The Netherlands), and printed using UltiMate™ polylactic acid (PLA+) filament (Hamilton, ON, Canada) on an Ultimaker S5 3D printer (Ultimaker B.V., Utrecht, The Netherlands). The silicone skin overlay was created by mixing 300 grams of Ecoflex™ 00-20 FAST silicone (Smooth-On, Macungie, PA, USA) with flesh-tone Silc-Pig™ coloring (Smooth-On), pouring the mixture into a 3D-printed mold, and allowing it to cure for 75 minutes. The overlay was then placed over the 3D-printed leg, tibia bone, and foam components, and positioned on the 3D-printed stand (Figure 1). The detailed development process of the IO infusion task trainer is described in another technical report [16].

**Figure 1:**
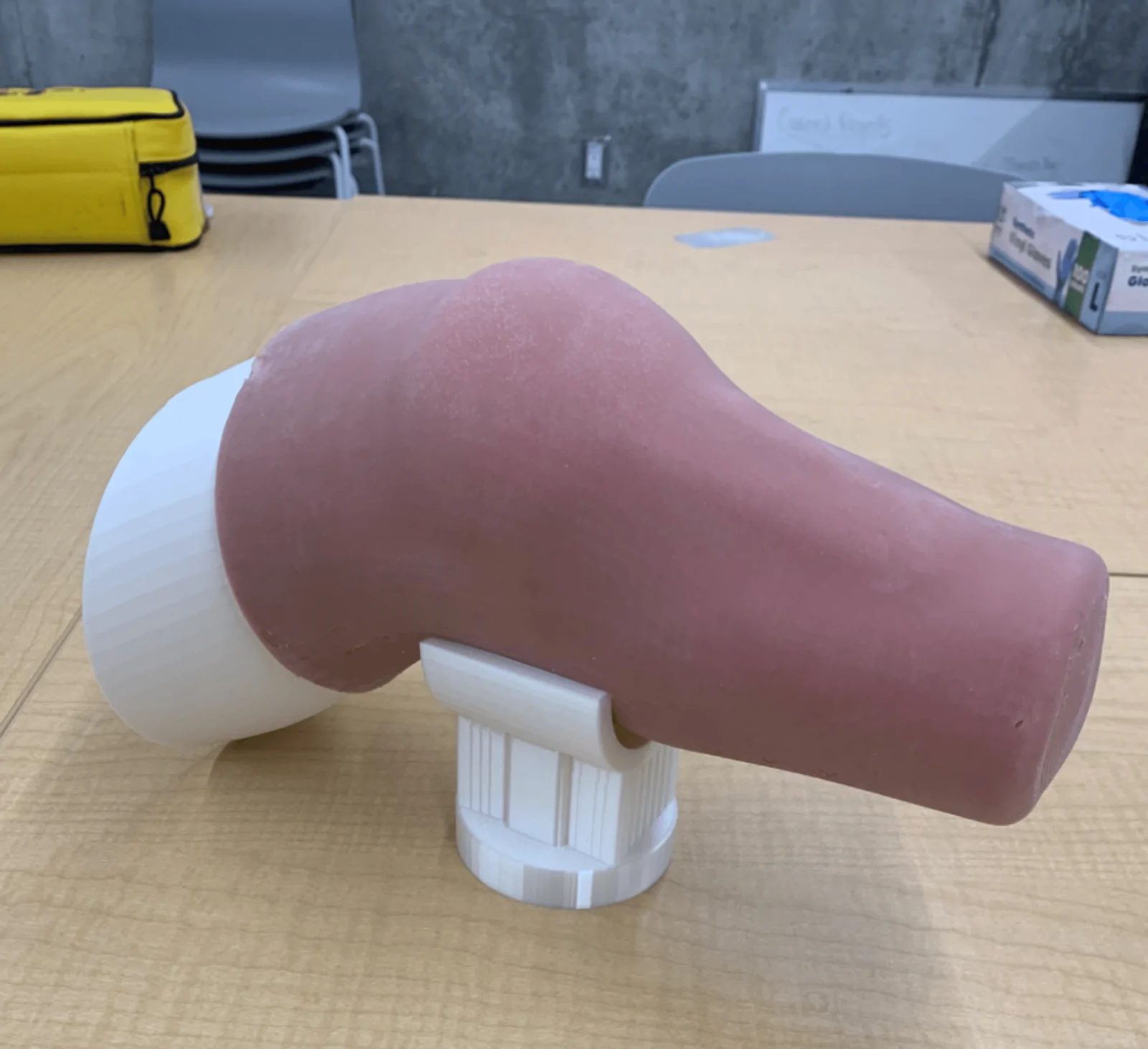
The IO infusion task trainer. IO, intraosseous.

For round 1, three IO infusion task trainers were assembled and used as the foundation for the three skin tear management task trainer prototypes.

To create the open wound, five grams of Ecoflex™ 00-35 FAST silicone (Smooth-On) and red Silc-Pig™ coloring (Smooth-On) were mixed until fully combined. The silicone was poured onto a flat sheet of clear plastic (MAYKU Form Box, Bermondsey, London, UK), allowed to spread into a thin, even layer, and left to cure for 10 minutes. While curing, a wound template, measuring 5 inches long and 3 inches wide, was cut from a second clear plastic sheet (MAYKU Form Box) (Figure 2). The template defined the desired wound shape. Once cured, the template was positioned on top of the silicone, and any excess silicone extending beyond the edges of the template was trimmed using a knife.

**Figure 2:**
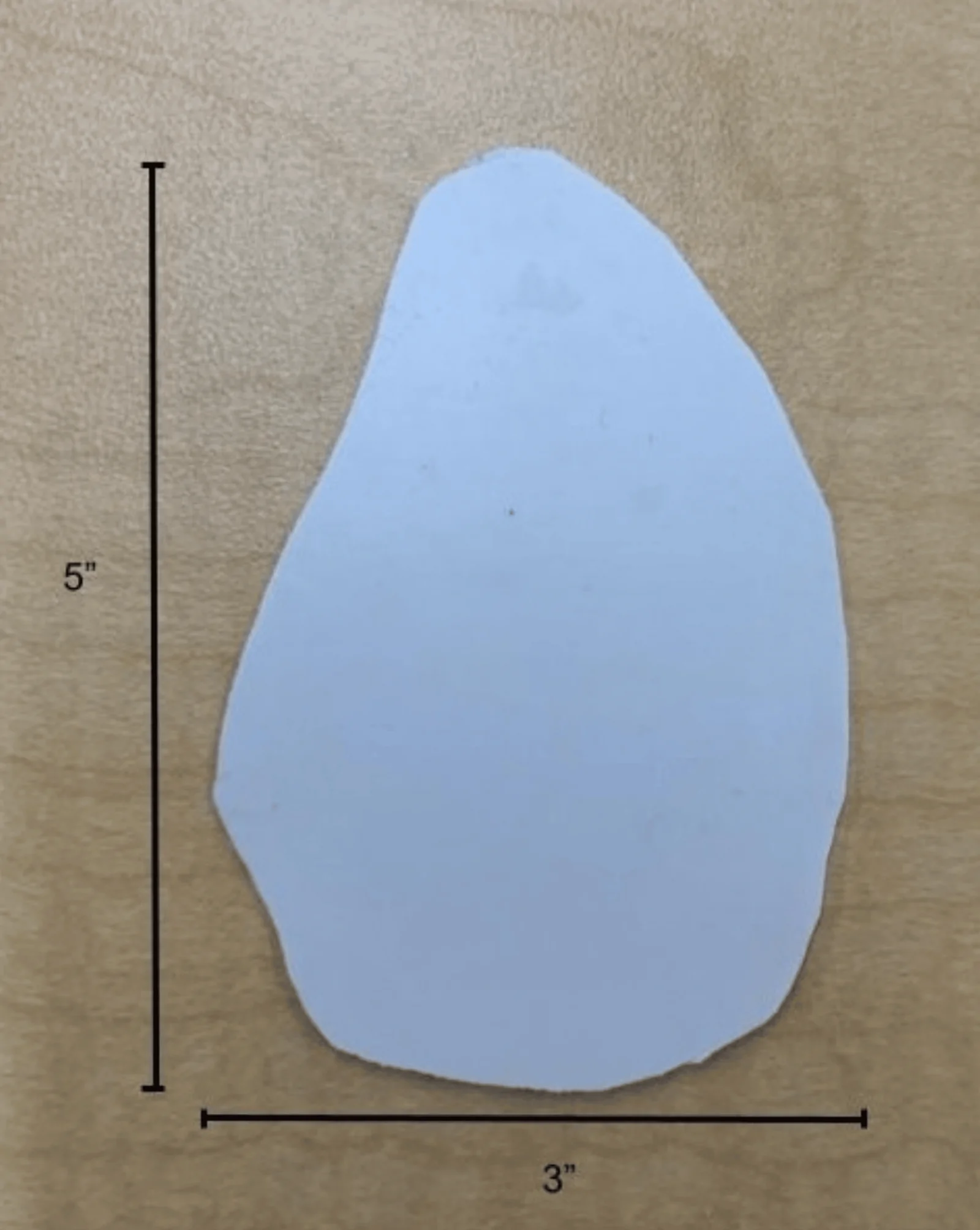
Wound template cut from the clear plastic sheet.

To create the skin flap, 15 grams of Ecoflex™ 00-35 FAST silicone (Smooth-On) and flesh-tone Silc-Pig™ coloring (Smooth-On) were mixed until fully combined. The silicone was poured over the wound template, and the plastic sheet was held at a downward angle to encourage spreading and achieve the desired thinness. After curing for five minutes, the skin flap was extracted from the template using a knife while maintaining a small attachment point between the flap and the open wound. The resulting prototype represented a type 1 skin tear (no skin loss). To simulate a type 2 skin tear (partial skin loss), a second prototype was created following the same procedure, but the flap was trimmed to expose a portion of the open wound. To simulate a type 3 skin tear, a third prototype was created in which the flap was fully removed from the wound.

To simulate dried blood, five grams of Ecoflex™ 00-35 FAST silicone (Smooth-On) and blood red Silc-Pig™ coloring (Smooth-On) were mixed until fully combined and applied around the open wounds. Each of the three skin tear prototypes was attached to an IO infusion task trainer and secured using 8 grams of Ecoflex™ 00-35 FAST silicone (Smooth-On) mixed with flesh-tone Silc-Pig™ coloring (Smooth-On).

Testing: Following testing, the clinical team determined that constraints 1 and 3, which focused on realism and adaptability, and optimizing costs, respectively, had been met. Analysis of the TAP session notes revealed that the prototypes were perceived as adequately realistic and suitable for supporting procedural skill acquisition and maintenance, confidence, and decision-making abilities. Additionally, the use of digital technologies and AM enabled rapid customization to represent different sizes, skin tones, and tear types, creating a versatile platform.

However, to further enhance realism, the clinical team suggested adding bruising around the wound and incorporating dirt that could be removed during the procedure. Further testing also revealed that the thin skin flaps on prototypes 1 and 2, while realistic, were too fragile to withstand repeated use. Both skin flaps detached from the wound when removing the dressing, meaning that these prototypes could not be reused for their intended purpose. Prototype 3 remained unaffected, as it did not include a skin flap. Thus, the second constraint, which focused on durability and reusability, was not satisfied for prototypes 1 and 2.

Round 2

Prototyping: Aligned with feedback from round 1, the objective of round 2 was to improve the durability of the skin flaps to enable repeated use of prototypes 1 and 2, while also enhancing the overall realism of all three prototypes. 

To achieve this objective, new skin flaps were created by cutting a square piece of power mesh (Green Brook, NJ, USA), measuring 6 inches long and 6 inches wide, and placing it flat on a clear plastic sheet (MAYKU Form Box). Ten grams of Ecoflex™ 00-35 FAST silicone (Smooth-On) and flesh-tone Silc-Pig™ coloring (Smooth-On) were mixed and poured over the power mesh, and a second plastic sheet (MAYKU Form Box) was placed over both the power mesh and silicone. Firm pressure was applied to the plastic sheet and maintained for five minutes to encourage the silicone to spread into a thin, even layer. Once cured, the plastic sheets were separated, and the mesh-reinforced silicone was removed. The wound template from round 1 was used to mark and cut two skin flaps from the mesh-reinforced silicone, leaving a small section of excess power mesh to enable attachment to the wound.

The two mesh-reinforced skin flaps were each positioned over an open wound. Two grams of Ecoflex™ 00-35 FAST silicone (Smooth-On) and flesh-tone Silc-Pig™ coloring (Smooth-On) were combined and poured over the excess power mesh to secure each flap to the wound. The resulting prototypes represented type 1 skin tears. To reflect a type 2 skin tear, the flap of one of the prototypes was trimmed to expose a portion of the open wound.

Two more IO infusion task trainers were developed for prototypes 1 and 2. Each wound and skin flap prototype was attached to an IO infusion task trainer and secured using eight grams of Ecoflex™ 00-35 FAST silicone (Smooth-On) mixed with flesh-tone Silc-Pig™ coloring (Smooth-On). Prototype 3 from round 1 was reused for round 2.

To simulate bruising, blood red and blue Silc-Pig™ colorings (Smooth-On) were applied around the wounds of all three prototypes using a Q-tip. The prototypes were allowed to dry for 24 hours and subsequently dusted with baby powder. Prior to testing, Aleene's Fabric Fusion Stiffen Quik Pump Spray Adhesive (ILoveToCreate, Los Angeles, CA, USA) was sprayed around the wounds of all three prototypes and beneath the flaps of prototypes 1 and 2, and dirt was applied around the wounds and beneath the flaps.

The approximate fabrication time per unit was 30 minutes of active labor and 90 minutes of curing for the type 1 and 2 skin tear prototypes. The type 3 skin tear prototype required 20 minutes of active labor and 80 minutes of curing, as this prototype did not require a skin flap attached to the open wound.

The three prototypes created in round 2 are shown in Figure 3.

**Figure 3:**
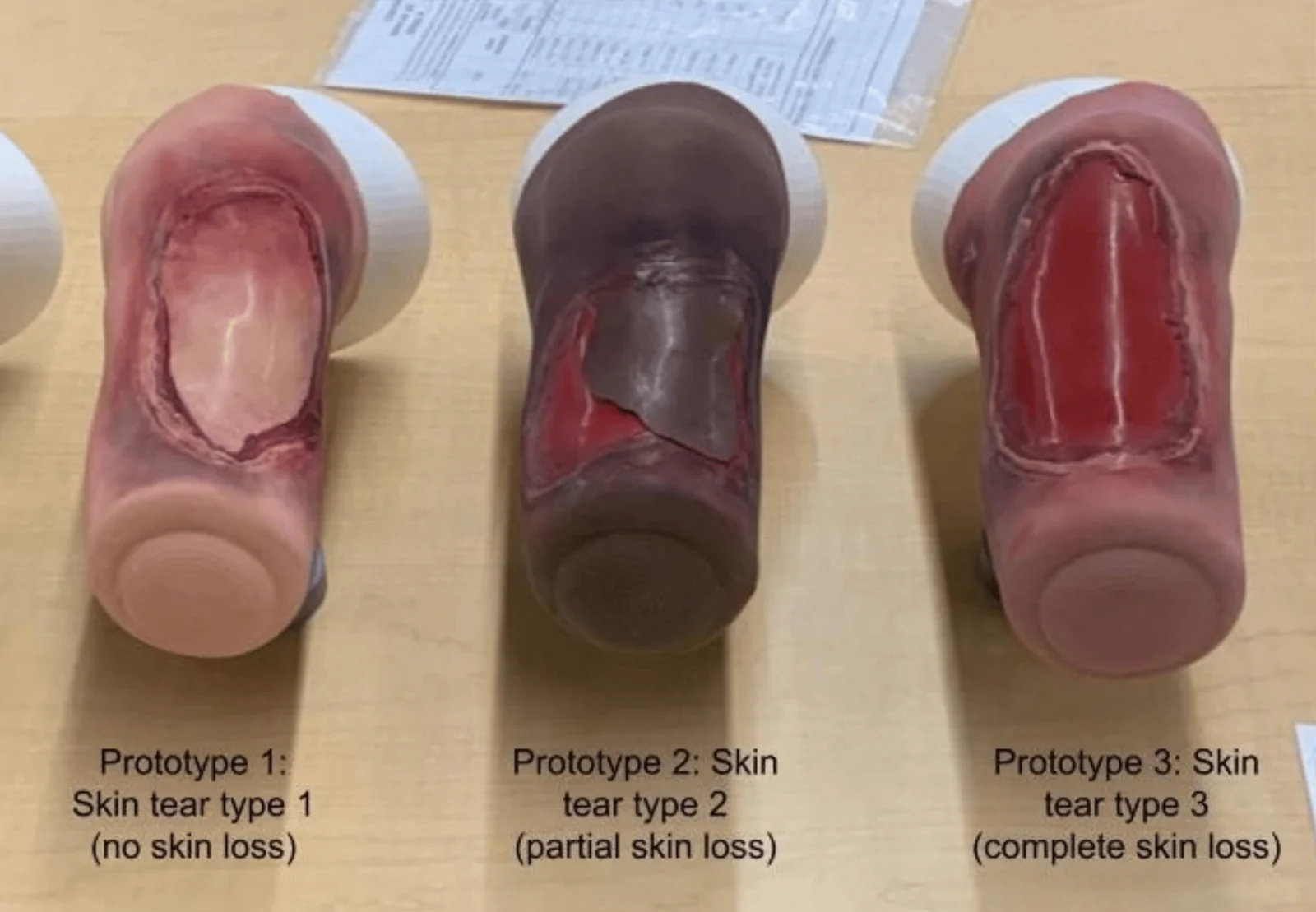
Prototypes 1, 2, and 3 developed in round 2.

Testing: Testing revealed that the mesh-reinforced skin flaps and simulated bruising remained intact after 37 complete cycles of irrigation, flap reapproximation, and dressing application and removal performed by the clinical team, supporting the repeated use of the task trainer. The use of Aleene's Fabric Fusion Stiffen Quik Pump Spray Adhesive (ILoveToCreate) allowed dirt to adhere to the wound surface and to be removed during the procedure. Following use, the wound could be easily cleaned to prevent adhesive residue from accumulating. The fabric stiffener adhesive also enabled the skin flaps to bunch together during manipulation, further enhancing realism.

Overall, the clinical team perceived the three prototypes as acceptable training tools and suitable for integration into SBE initiatives. However, they suggested developing task trainers with varying skin flap thicknesses to better reflect diverse patient populations.

Assessment of the Skin Tear Management Task Trainer

For assessment purposes, the clinical team recommended using only prototype 1 (type 1 skin tear; no skin loss), as this task trainer would allow participants to practice all components of skin tear management, including wound cleaning and irrigation, skin flap reapproximation, and dressing application. Additionally, in response to feedback from round 2, skin flaps were created in varying thicknesses by increasing or decreasing the pressure applied to the plastic sheet and mesh-reinforced silicone during fabrication. Increased pressure resulted in thinner skin flaps. Fourteen type 1 skin tear management task trainers representing various skin tones and flap thicknesses were developed by the research team and integrated into the training pathway created by RDPS (Figure 4). Since skin flap thickness could not be quantified, it was not recorded at an individual level, and participants were not systematically allocated to a specific flap thickness.

**Figure 4:**
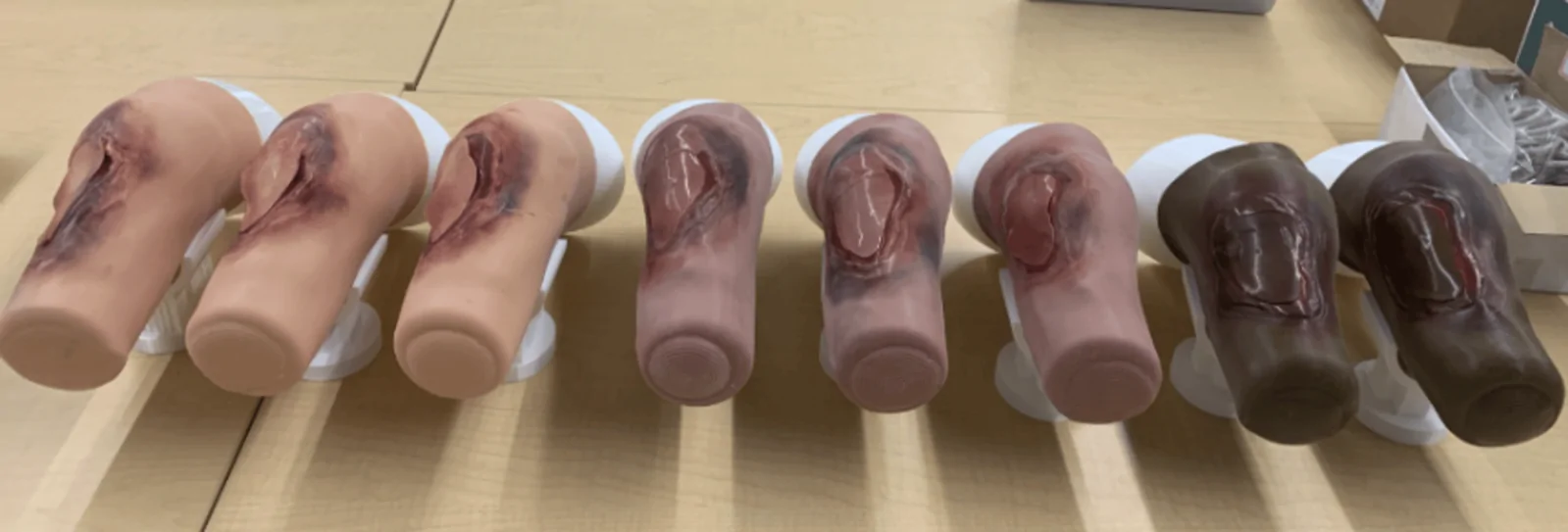
A set of type 1 skin tear management task trainer prototypes developed by the research team.

Data collection followed a mixed-methods approach, integrating both qualitative and quantitative methods [17]. A survey incorporating five-point Likert scales, multiple-choice questions, and open-ended questions was developed in Google Forms (Google), following a modified version of the Michigan Standard Simulation Experience Scale (MiSSES) template [18]. Consistent with the intended adaptability of the MiSSES template, questions were selected and modified to focus specifically on the task trainer rather than the broader training session or curriculum [18]. The teaching quality domain was excluded as instructor performance and instructional resources were outside the scope of the present work; the fidelity domain was operationalized as realism, and item wording was modified where necessary to refer specifically to the task trainer rather than the broader training pathway. The original five-point Likert scale was retained. The survey consisted of 15 questions across three domains: (1) self-efficacy, (2) realism, and (3) educational value. The 15 questions that encompassed the survey are detailed in Table 2.

**Table 2:** Survey questions used to assess the skin tear management task trainer. N/A, not applicable.

Domain	Question number	Question type	Question
Self-efficacy	1	Likert scale	The task trainer helped improve my competence (knowledge + skills + attitudes) in managing skin tears.
	2	Likert scale	The task trainer helped improve my confidence in managing skin tears.
	3	Likert scale	The task trainer helped improve my ability to manage skin tears independently.
	4	Open-ended	Comments regarding the task trainer that may improve your self-efficacy.
Realism	5	Likert scale	The task trainer had adequately realistic characteristics and features.
	6	Likert scale	The task trainer felt accurate.
	7	Likert scale	The task trainer was durable.
	8	Likert scale	I was able to successfully perform skin tear management-related skills (reapproximating skin, wound cleaning, dressing application, etc.) using this task trainer.
	9	Open-ended	Comments/suggestions to improve the realism of the task trainer.
Educational value	10	Likert scale	The task trainer is a good training tool for increasing knowledge in managing skin tears.
	11	Likert scale	The task trainer is a good training tool for developing skills in managing skin tears.
	12	Open-ended	Comments/suggestions to improve the educational value of the task trainer.
General	13	Multiple choice	GLOBAL: Please check one statement below with which you most agree: (A) This task trainer requires extensive improvements before it can be considered for use in training. (B) This task trainer requires minor adjustments before it can be considered for use in training. (C) This task trainer should be improved slightly, but it can be used in training. (D) The task trainer requires no improvements and can be used in training.
	14	Open-ended	Please suggest any changes you would make to the task trainer to improve your learning experience.
	15	Open-ended	What alternative task trainers have you used to train this skill, and how does it compare to this task trainer? If no alternative was used, please write N/A.

Questions 1, 2, 3, 5, 6, 7, 8, 10, and 11 required participants to rate the task trainer according to a five-point Likert scale, where one represented low levels of agreement, and five indicated high levels of agreement. Question 13 was a multiple-choice question that asked participants to select the extent to which improvements should be made before the task trainer can be used in training, while questions 4, 9, 12, 14, and 15 were open-ended questions designed to gather broad insights on the task trainer’s perceived acceptability.

After completing the didactic component of the RDPS training pathway and participating in hands-on practice using the task trainer, participants were asked to complete the survey to provide feedback. All survey responses were exported to Microsoft Excel (Microsoft Corporation, Redmond, WA, USA) for statistical analysis. Given the ordinal nature of the Likert-scale data, response frequencies and percentages, means, standard deviations (SDs), medians, and interquartile ranges (IQRs) were calculated descriptively and interpreted for questions 1, 2, 3, 5, 6, 7, 8, 10, and 11. A mean score of ≥ 3.5 and SD ≤ 1.0 was considered indicative of perceived acceptability. This threshold is consistent with best practices reported in the literature on consensus-building methodologies [19]. For the multiple-choice question (question 13), ordinal data were collected and analyzed by calculating the number of participants selecting each response as a percentage of the total number of respondents.

Responses to open-ended questions (questions 4, 9, 12, 14, 15) were reviewed by one member of the research team (AJ) to identify recurring patterns and opportunities for refinement. Similar responses were grouped into descriptive categories and representative quotations were selected to illustrate each. As AJ was also involved in the development of the task trainer, these findings were interpreted as descriptive qualitative feedback, and the potential for researcher interpretation bias was acknowledged. Saturation was not assessed because the open-ended responses were collected as part of a fixed survey sample rather than through an iterative qualitative sampling process.

Qualitative and quantitative data were used to determine the perceived acceptability of the task trainer and its suitability for integration into SBE training initiatives.

Outcome

Development of the Skin Tear Management Task Trainer

The total cost of the materials used to develop the task trainer was 35.29 CAD, including taxes. The costs are broken down in Table 3. This includes the costs of the previously developed IO task trainer, as its components were repurposed as the foundation for the skin tear management task trainer [16]. The cost of the mold for the silicone overlay and the wound template was not included, as these components can be reused to produce multiple task trainers. It is important to note that the cost of the wound and skin flaps is approximate, as it is difficult to estimate the amount of power mesh-reinforced silicone cut from the larger sheet. Additionally, the exact cost may differ slightly between individual skin tear management task trainers, as the thinner skin flaps will require less mesh-reinforced silicone. Moreover, this cost reflects only the task trainer with a type 1 skin tear. Types 2 and 3 (partial skin loss and total skin loss) are expected to have marginally lower costs, as less mesh-reinforced silicone will be required. However, these differences are unlikely to affect accessibility or scalability.

**Table 3:** Cost breakdown of the materials used to develop the skin tear management task trainer with a type 1 skin tear. CAD, Canadian dollars; IO, intraosseous.

Component	Material	Amount used	Cost in CAD (including taxes)
Adult IO infusion leg	UltiMate™ polylactic acid (PLA+)	690 grams	20.15
Adult tibia bone	UltiMate™ polylactic acid (PLA+)	95 grams	2.78
Fat	Foam	10 x 6 inch oval-shaped piece	1.31
Skin overlay	Ecoflex™ 00-20 FAST silicone	300 grams	10.05
Wound and skin flap	Ecoflex™ 00-35 FAST silicone and Power Mesh	15 grams silicone and 6 x 6 inch piece of Power Mesh	1.00
			Total cost = 35.29

Assessment of the Skin Tear Management Task Trainer

Of the 471 frontline and community paramedics who participated in the RDPS training pathway, a total of 61 completed the survey anonymously (response rate of 12.95%). Participants included both primary and advanced care paramedics with years of experience ranging from one month to 36 years.

The quantitative data showed that the task trainer received an overall mean score of 4.7 on a five-point Likert scale. Feedback was categorized into three domains: (1) self-efficacy, (2) realism, and (3) educational value. Mean scores and SDs were calculated for each question that used a Likert scale (questions 1, 2, 3, 5, 6, 7, 8, 10, and 11), as well as mean scores for each domain. Overall, the task trainer achieved mean scores of ≥ 3.5 and SD ≤ 1.0 across all questions, indicating that participants perceived it as an acceptable training tool [19]. In the self-efficacy domain (questions 1-3, with question 4 open-ended), the task trainer received a mean score of 4.68 on a five-point Likert scale, which suggests that participants felt it improved their confidence and ability to manage skin tears independently. In the realism domain (questions 5-8, with question 9 open-ended), the task trainer received a mean score of 4.64 on a five-point Likert scale, suggesting that participants perceived it as adequately realistic. Lastly, in the educational value domain (questions 10-11, with question 12 open-ended), the task trainer received a higher mean score of 4.85 on a five-point Likert scale, indicating that participants perceived it as a useful training tool for developing skin tear management procedural skills.

Questions 5 and 6 each received only 60 responses, as one participant did not provide a response to these questions. Accordingly, descriptive statistics for these questions were calculated using a denominator of 60, yielding mean scores of 4.53 and 4.55, respectively. Additionally, medians and IQRs were calculated for each question. Responses were concentrated toward the upper end of the scale, with all questions receiving medians of 5 and IQRs from 4-5 to 5-5. This was most pronounced for question 11, for which 55 of 61 participants (90.16%) selected the maximum score of 5, resulting in a median of 5 and an IQR of 5-5. The Likert scale response distributions and percentages, means, SDs, medians, and IQRs are presented in Table 4. Questions 4 (self-efficacy domain), 9 (realism domain), and 12 (education value domain) were open-ended and did not have quantitative results. Hence, they have been omitted from Table 4.

**Table 4:** Quantitative survey data for the skin tear management task trainer. SD, standard deviation; IQR, interquartile range.

Skin tear management task trainer quantitative survey data											
Domain	Question number	Likert scale frequencies, n (%)					Number of respondents	Mean	SD	Median	IQRs
		1	2	3	4	5					
Self-efficacy	1	0 (0%)	1 (1.64%)	2 (3.28%)	13 (21.31%)	45 (73.77%)	61	4.67	0.62	5	4-5
	2	0 (0%)	1 (1.64%)	3 (4.92%)	13 (21.31%)	44 (72.13%)	61	4.64	0.66	5	4-5
	3	0 (0%)	1 (1.64%)	1 (1.64%)	12 (19.67%)	47 (77.05%)	61	4.72	0.58	5	5-5
Realism	5	0 (0%)	2 (3.33%)	2 (3.33%)	18 (30%)	38 (63.33%)	60	4.53	0.92	5	4-5
	6	1 (1.67%)	1 (1.67%)	2 (3.33%)	16 (26.67%)	40 (66.67%)	60	4.55	0.98	5	4.5-5
	7	0 (0%)	1 (1.64%)	2 (3.28%)	12 (19.67%)	46 (75.41%)	61	4.69	0.62	5	5-5
	8	0 (0%)	1 (1.64%)	2 (3.28%)	7 (11.48%)	51 (83.61%)	61	4.77	0.59	5	5-5
Educational value	10	0 (0%)	0 (0%)	1 (1.64%)	10 (16.39%)	50 (81.97%)	61	4.80	0.44	5	5-5
	11	0 (0%)	0 (0%)	0 (0%)	6 (9.84%)	55 (90.16%)	61	4.89	0.44	5	5-5

Responses to the multiple-choice question revealed that 34 (55.7%) participants agreed that the task trainer could be used in SBE training initiatives with no improvements. A total of 21 (34.4%) participants indicated that the task trainer should be improved slightly, but could be used in training. Three (4.9%) participants stated that the task trainer required minor improvements before it could be considered for use in training, and another three (4.9%) participants stated that extensive improvements were required. The results of the multiple-choice question are shown in Figure 5.

**Figure 5:**
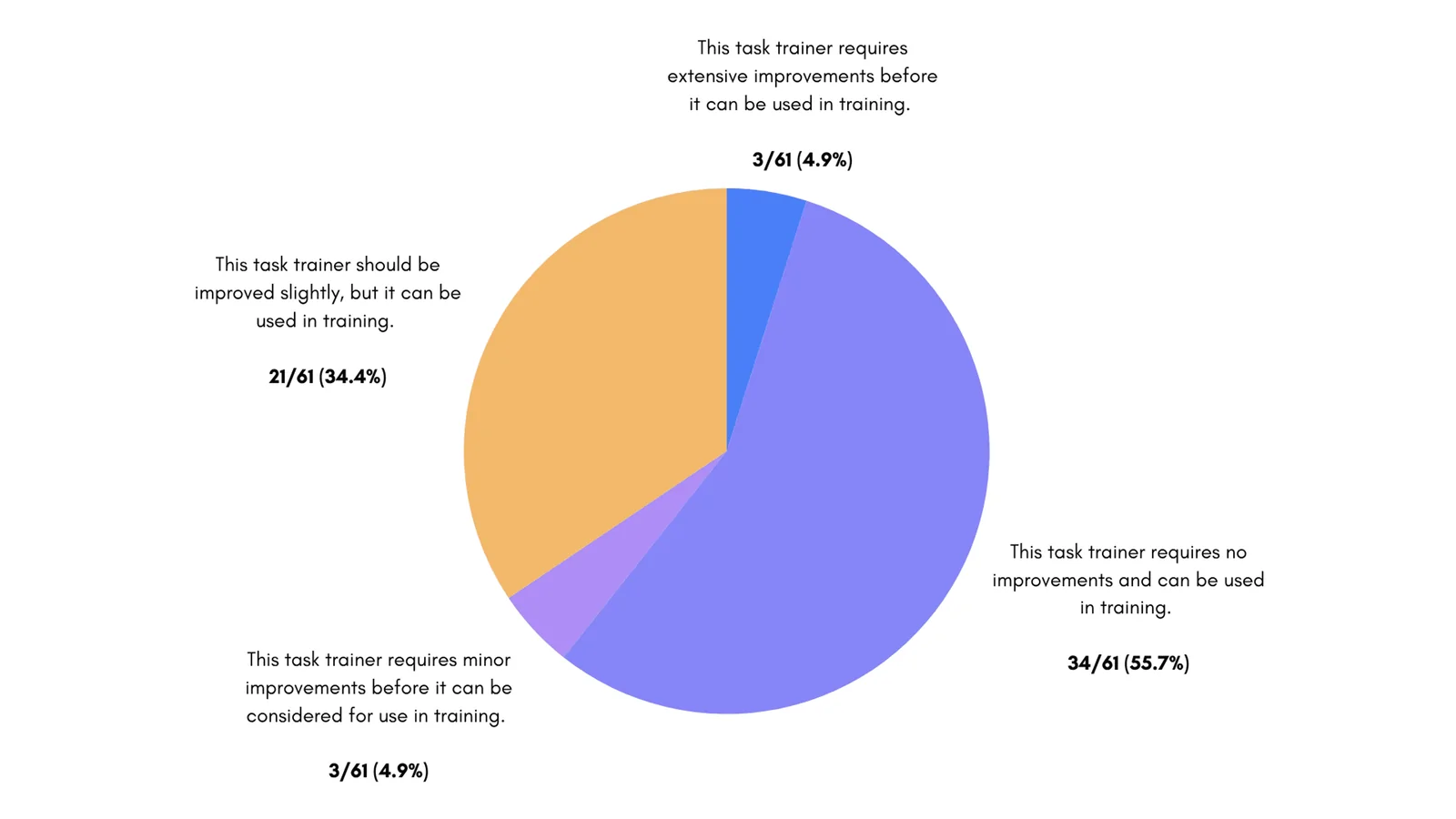
Pie chart showing the results of the multiple-choice question.

Qualitative data revealed that participants generally perceived the task trainer as an acceptable training tool and a beneficial addition to the RDPS training pathway. Notably, in question 15, all respondents indicated that they had not previously used any alternative task trainers to practice skin tear management, highlighting the limited availability of training tools within this educational context.

Three descriptive categories were identified through thematic analysis of the open-ended question responses: (1) visual and tactile realism, (2) pathological realism, and (3) procedural complexity and adaptability. Category 1, visual and tactile realism, refers to the properties of the materials and manufacturing processes used to develop the task trainer and their influence on realism. Participants appreciated the overall realism of the task trainer but noted that the skin flaps lacked the adhesion and elasticity of human skin, limiting their ability to naturally bunch and fold during manipulation. Additionally, some participants suggested that the skin flaps should be thinner and more malleable to better replicate skin tears observed in human patients, noting that task trainers with the thinnest skin flaps were perceived as the most realistic. However, since participants were not systematically allocated to a specific flap thickness and thickness was not recorded at the individual level, the association between flap thickness and perceived realism could not be analyzed and is reported as a qualitative observation only. Participants also recommended incorporating rougher edges around the wound and skin flap to further enhance realism.

Category 2, pathological realism, refers to the wound-related characteristics that contribute to the authenticity of the simulated experience. Participants appreciated the addition of dirt and bruising, but suggested incorporating simulated blood or fluids between skin layers to further enhance realism and represent a broader range of wound presentations.

Category 3, procedural complexity and adaptability, focuses on the potential for the task trainer to be adapted to adjust procedural complexity, represent diverse clinical presentations, and accommodate learner- and program-specific needs. Participants appreciated the inclusion of diverse skin tones and the ability to modify the skin flap to represent different skin tear types. However, they suggested incorporating additional clinical presentations (e.g., dehydrated skin) to further increase the utility of the task trainer. Participants also recommended developing task trainers with skin tears located on different anatomical sites (e.g., hinge joints and elbows) to better reflect diverse clinical scenarios. Additionally, participants suggested creating task trainers of varying sizes, with skin tears of diverse shapes, sizes, and levels of procedural difficulty, to support scalability across learner levels and educational programs.

Table 5 lists the identified descriptive categories and supporting quotes derived from the qualitative analysis of the open-ended question responses.

**Table 5:** Summary of descriptive categories and supporting quotes derived from the qualitative analysis of the open-ended question responses.

Skin tear management task trainer qualitative survey data	
Descriptive category	Supporting quotes
		Visual and tactile realism	“Find a material that is more similar to skin as the silicone flaps don't act the same due to their thickness and lack of adhesion” (Participant 12). “Ability to fold skin flap onto itself better” (Participant 14). “The skin flaps are heavy and floppy, possibly make them lighter?” (Participant 15). “Lighter more malleable skin flaps” (Participant 15). “Thinner skin flap to make it more realistic. But I also understand durability for long term use will be a challenge with making the flap thinner as they tend to be that way on patients” (Participant 16). “It’s hard for skin reapproximation, however this is understandable since it’s hard to recreate the elasticity of skin” (Participant 31). “Rougher edges of the skin, it often doesn’t have such clean edges” (Participant 50).
Pathological realism	“Maybe have some fake blood to simulate the whole process” (Participant 13). “Create more life like skin layers with bleeding and fluids” (Participant 29). “Wet blood, pus” (Participant 43). “Ability to have blood or other fluids added” (Participant 46). “Have it be able to “bleed, seep” for realism and cleaning” (Participant 47).
Procedural complexity and adaptability	“Would like to try skin flap rehydration” (Participant 14). “Different presentation of wounds (i.e., dehydrated)” (Participant 22). “Practice using all materials in more spots (leg, head, arms)” (Participant 23). “Make it more difficult to re-approximate skin” (Participant 28). “Different size/type of skin tears” (Participant 34). “Other areas that may be different or difficult to address” (Participant 46). “Different body parts” (Participant 50). “More variety of areas for skin tears (hands, hinge joints)” (Participant 57). “It would be cool if you could make a weeping wound” (Participant 61)

## Discussion

This technical report described the development and initial assessment of a scalable, low-cost skin tear management task trainer for paramedic education. The task trainer was co-developed through collaboration between researchers and clinicians, ensuring that design decisions were informed by both technological expertise and the clinical and operational realities of prehospital care. The task trainer received an overall mean score of 4.7 out of five, with all Likert scale survey questions achieving mean scores ≥ 3.5 and standard deviations ≤ 1.0, indicating that frontline and community paramedics generally perceived it as an acceptable training tool.

Although participants rated the task trainer highly across the self-efficacy, realism, and educational value domains, responses to question 13 revealed that 44.3% of participants identified some degree of room for improvement. This does not necessarily conflict with the high Likert scale ratings, as the majority of participants (34.4%) indicated that only slight refinement was needed and that the task trainer could still be used for training. Qualitative feedback further clarified that these suggestions primarily concerned refinements to realism, wound presentation, and procedural complexity rather than fundamental concerns about the trainer’s suitability for use.

The combination of realism, low-cost, durability, and adaptability positions this task trainer as a scalable solution for diverse paramedic education programs and academic training pathways. The use of digital technologies and AM enabled low-cost production while maintaining realism, adaptability, and durability, adhering to the design-to-cost approach [14]. The use of this approach addressed barriers commonly associated with commercially available task trainers, such as the skin tear with flap developed by Techline Trauma, priced at 146.61 CAD [10], which may be expensive, limited in anatomical realism, and inflexible in design. Consequently, the task trainer described in the technical report has the potential to facilitate broader adoption of SBE initiatives, particularly in rural, remote, and resource-constrained settings.

A key innovative feature of this task trainer is its modular architecture, which enables the rapid customization of characteristics, including size, skin tone, skin flap type and thickness, and wound characteristics. This adaptability allows educators to modify the task trainer to reflect diverse patient populations and align with learner and program-specific objectives, supporting SBE for novice to advanced learners. For example, the design can be scaled to 45% of its full size to create a pediatric skin tear management task trainer, allowing learners to progress to smaller, more intricate procedural skills as they gain proficiency and confidence (Figure 6).

**Figure 6:**
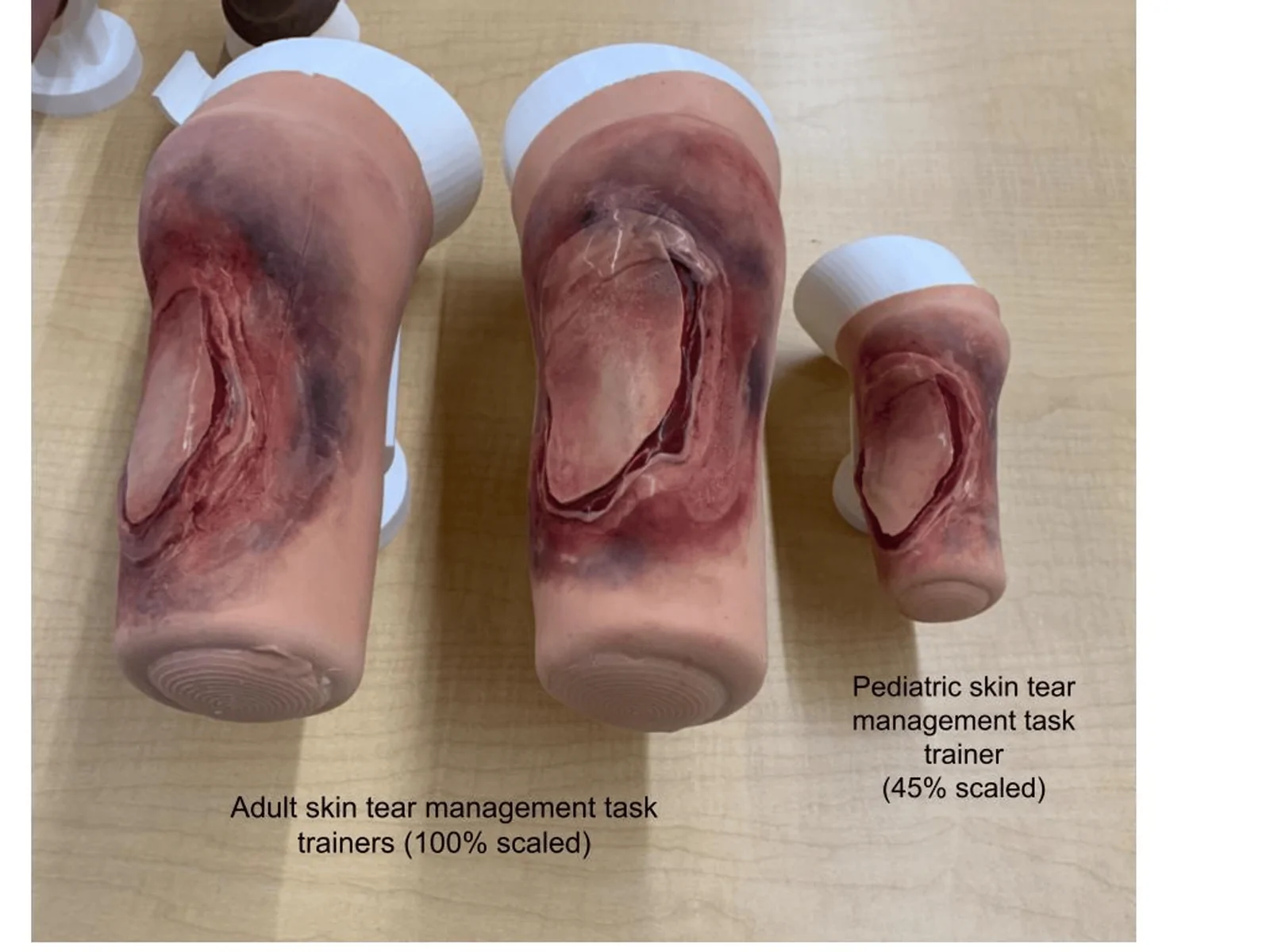
Adult skin tear management task trainers (left and center, 100% scaled) and pediatric skin tear management task trainer (right, 45% scaled).

Additionally, the circular bottom of the skin tear overlay can be easily cut and removed, allowing the overlay to be applied directly to the leg of a standardized patient (Figure 7). This approach provides an opportunity to transition from isolated skills practice toward more immersive simulation experiences for advanced learners.

**Figure 7:**
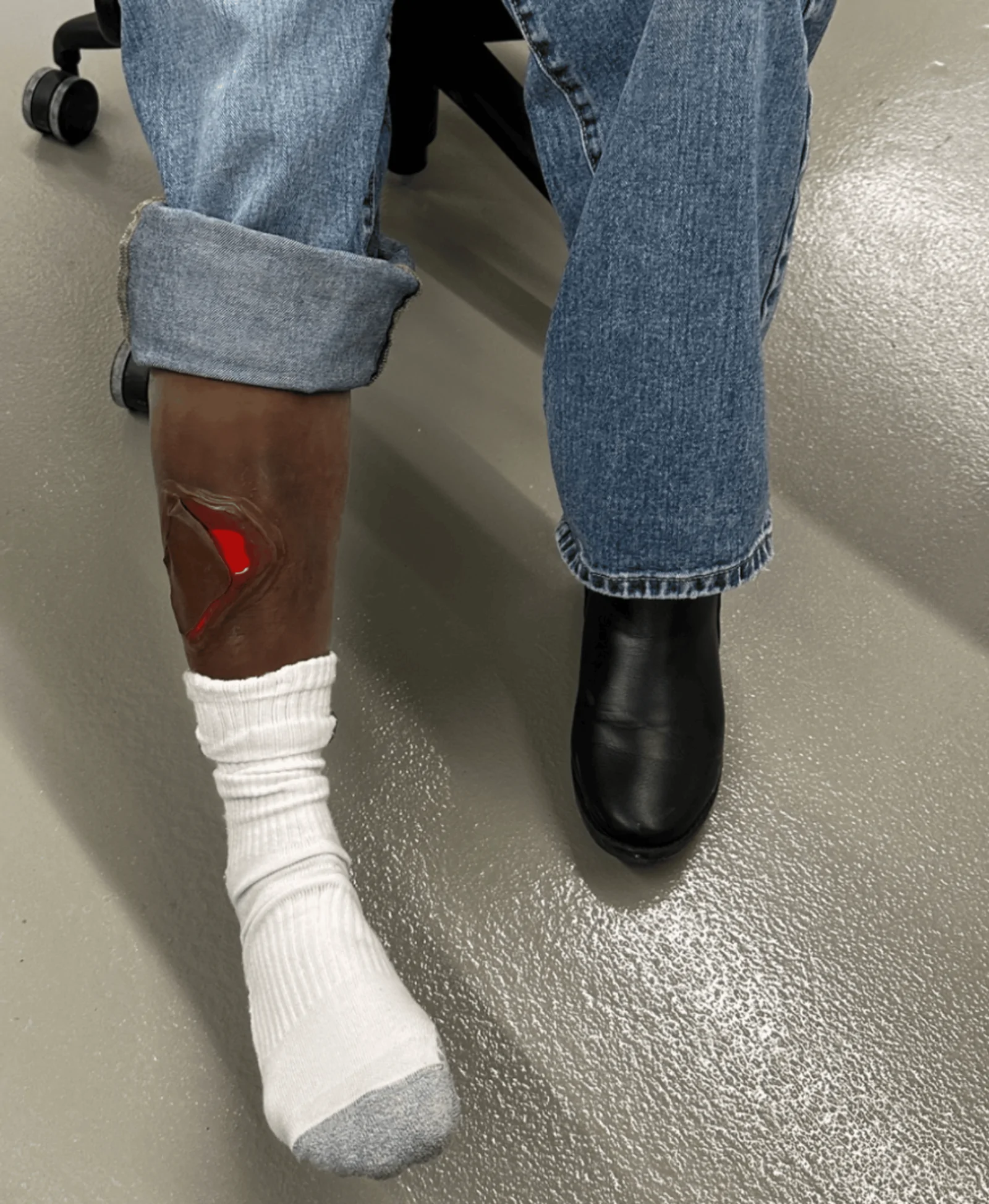
Skin tear overlay applied directly to the leg of a standardized patient.

Furthermore, the task trainer was developed using components from a previously developed adult IO infusion task trainer [16], allowing a single task trainer to support training for multiple procedural skills. This approach may maximize educational value while reducing the resources required to develop, store, and maintain separate task trainers.

Participant feedback also identified opportunities for continued refinement of the task trainer. Notably, they indicated that the thinner skin flaps were the most realistic. Additionally, participants suggested increasing the adhesion and malleability of the skin flaps and developing task trainers with additional wound characteristics (e.g., blood or fluids) to represent a broader range of clinical presentations, as well as task trainers with skin tears in varying anatomical locations.

Limitations

This technical report has several limitations. First, assessment relied on self-reported perceptions of self-efficacy, realism, and educational value collected immediately after a single hands-on session, rather than pre-and post-training or performance-based measures of procedural skill acquisition and maintenance, confidence, and decision-making abilities. As such, this report could not determine the educational effectiveness of the task trainer or skin tear management training pathway created by RDPS. Second, all participants came from a single paramedic service, limiting the generalizability of the findings. Third, no participants had used an alternative skin tear task trainer, which constrained their ability to reference and compare this task trainer with past hands-on skin tear management training experiences. Fourth, ceiling effects were evident across several Likert-scale items, particularly within the educational value domain. This concentration of responses at the upper end of the scale limits the instrument’s ability to discriminate between participants with favorable and very favorable perceptions of the task trainer. Fifth, social desirability bias also cannot be excluded, as the task trainer was assessed within participants’ own organizational curriculum. Sixth, skin flap thickness was neither controlled nor recorded at the individual level, as the fabrication process relied on manually applying pressure to the plastic sheet and mesh-reinforced silicone, making it difficult to quantify. As a result, the association between flap thickness and perceived realism is reported as a qualitative observation only. Seventh, this work concluded that the mesh-reinforced skin flaps and simulated bruising remained intact after 37 complete cycles of irrigation, flap reapproximation, and dressing application and removal performed by the clinical team; however, the durability of these components beyond 37 cycles remains unknown. Lastly, only the task trainer representing a type 1 skin tear was assessed through this work; therefore, limitations associated with using the task trainer to practice management of type 2 and type 3 skin tears may not have been captured.

Future directions

Subsequent developments will focus on standardizing the manufacturing process to improve consistency between task trainers. Digital technologies and AM will be used to optimize skin flap thinness, malleability, and adhesion, incorporate additional wound characteristics and clinical presentations, and develop task trainers representing a broader range of anatomical locations. These modifications may further enhance the versatility of the task trainer and increase its scalability across diverse training initiatives.

Future research will focus on refining the task trainer and subsequently integrating it broadly across SBE initiatives and formally evaluating its impact on procedural skill acquisition and maintenance, confidence, and decision-making abilities among paramedics, as well as examining its utility and scalability in other health professions education in which practitioners may perform skin tear management, wound care, and IO infusion procedures.

## Conclusions

This technical report demonstrates the feasibility of developing a scalable, low-cost skin tear management task trainer for paramedic education using digital technologies and AM. Through a collaborative, iterative design process, this task trainer was developed to address key barriers associated with commercially available options, supporting broader scalability. Initial assessment indicated that the task trainer was perceived as an acceptable training tool and suitable for integration in SBE initiatives. Future work will focus on standardizing skin flap thinness and manufacturing processes, expanding the range of clinical presentations, and developing task trainers with skin tears in varying anatomical locations, followed by integrating the task trainer broadly across training initiatives and formally evaluating its impact on procedural skill acquisition and maintenance, confidence, and decision-making abilities.
